# Barriers to and facilitators of deprescribing for older people in secondary care in Saudi Arabia: a qualitative study using a theory-based approach

**DOI:** 10.1186/s12877-026-07486-8

**Published:** 2026-04-14

**Authors:** Turkeah M. Alenzy, Heather E. Barry, Saad A. Alkahtani, Carole Parsons

**Affiliations:** 1 School of Pharmacy, Queen’s University Belfast, 97 Lisburn Road, Belfast, BT9 7BL UK; 2 Department of Clinical Pharmacy, College of Pharmacy, Princess Nourah bint Adulrahman University, Riyadh, Saudi Arabia; 3 Department of Clinical Pharmacy, College of Pharmacy, Najran University, Najran, Saudi Arabia

**Keywords:** Potentially inappropriate medication, Deprescribing, Older people, Behaviour change, Hospital settings

## Abstract

**Background:**

In Saudi Arabia, over 55% of older people are exposed to potentially inappropriate medications. Deprescribing, a structured process to identify and discontinue unnecessary or harmful medications under medical supervision, by healthcare professionals (physicians and pharmacists) remains limited. Understanding barriers to and facilitators of deprescribing for older people from the perspectives of these healthcare professionals is essential for developing effective hospital-based deprescribing interventions.

**Methods:**

Semi-structured interviews were conducted with physicians and pharmacists working in hospitals in southern Saudi Arabia, recruited through purposive sampling. Eligible participants were physicians who worked with older inpatients and pharmacists with a ward-based role who had input into prescribing decisions. Theoretical Domains Framework (TDF2)-based interviews were conducted until data saturation was achieved. Inductive reflexive thematic analysis was first performed to generate themes from the data. Themes representing barriers and facilitators were then deductively mapped to TDF2 domains to prioritise domains and identify relevant behaviour change techniques (BCTs).

**Results:**

Twenty physicians and 20 pharmacists were interviewed. Six prioritised TDF2 domains represented determinants for deprescribing: *Social/professional role and identity*, *Social influences*, *Environmental context and resources*, *Knowledge*, *Behavioural regulation*, and *Beliefs about consequences*. Key barriers included pharmacists’ limited role, clinicians’ perceptions of negative patient and carer attitudes toward deprescribing, concerns about negative outcomes, lack of guidelines and documentation, and resource constraints. Facilitators included inter-professional support, clinician education, and recognition of deprescribing benefits. Forty BCTs were identified for inclusion in a deprescribing intervention.

**Conclusion:**

This study provides a foundation for designing a theory-informed intervention to enable deprescribing in Saudi hospital settings. It highlights context-specific influences, including the need to enhance coordination across care levels and improve systemic supports for deprescribing.

**Supplementary Information:**

The online version contains supplementary material available at 10.1186/s12877-026-07486-8.

## Background

Polypharmacy, defined as the concurrent use of multiple medications [1], has no universally agreed numerical threshold, although it is commonly defined as the use of five or more medications [2]. Increasingly, a distinction is made between ‘appropriate polypharmacy’, (the use of clinically necessary, evidence-based medications), and ‘inappropriate polypharmacy’, where the number or type of medications causes risks or provides limited benefit to patients [2, 3]. In this context, deprescribing, defined as the supervised process of withdrawing inappropriate medications to reduce harm and improve outcomes [4], has emerged as a targeted intervention.

Polypharmacy is especially prevalent among older people [5] and is strongly associated with use of potentially inappropriate medications (PIMs), where risks outweigh benefits [6]. In Saudi Arabia, the prevalence of PIMs among older people in hospital settings is reported at 57.2–63.6%, with polypharmacy and multimorbidity the main drivers [7, 8].

Despite growing awareness of the risks associated with PIMs, deprescribing is not yet effectively implemented in routine practice [9]. Challenges arise when implementing deprescribing at patient, healthcare professional (HCP), and healthcare system levels. At the patient level, limited health literacy and strong beliefs about the necessity of long-term medications reduce acceptance of deprescribing [10, 11]. At the HCP level, the complexity of multimorbidity and polypharmacy complicate assessment and medication review. Time pressures, clinical inertia, limited training, and reluctance to change treatments initiated by other HCPs are additional barriers [12, 13]. At the healthcare system level, fragmented care, poor interprofessional communication, and lack of access to comprehensive medical records further inhibit deprescribing practices [14, 15].

While previous studies have explored HCPs’ perspectives on deprescribing, much of the evidence has focused on primary and long-term care settings [15]. Although hospital-based research is increasing [16–22], deprescribing in hospitals remains complex because the prioritisation of acute treatment often deprioritises deprescribing decisions [15, 16]. Despite an improved understanding of general deprescribing barriers, translating this knowledge into routine practice remains a challenge. Addressing this persistent research-to-practice gap requires coordinated action across multiple socio-ecological levels [23], which necessitates a deeper understanding of specific behavioural influences that shape deprescribing decisions across professional roles and organisational contexts [24]. Regarding the local context, studies investigating these dynamics in Saudi Arabia remain scarce. Existing local research has either focused on physicians’ general knowledge [17] or perspectives in isolation [20]. Consequently, a theory-based exploration involving both physicians and pharmacists is essential to capture the collaborative nature of deprescribing. As local determinants may differ from those reported internationally, identifying these specific influences is critical to inform practical theory-based implementation strategies [24]. The Theoretical Domains Framework version 2 (TDF2) offers a structured approach to assess these behavioural determinants across 14 domains [25, 26].

## Methods

### Study design and setting

This qualitative study employed one-to-one semi-structured interviews using TDF2-based topic guides to explore the perspectives of physicians and pharmacists on the factors influencing deprescribing (barriers and facilitators) for older people in hospitals in Saudi Arabia. The TDF domains relevant to the barriers and facilitators of deprescribing were prioritised and mapped to BCTs to inform the development of a future intervention. This study is reported in accordance with the Consolidated Criteria for Reporting Qualitative Research (COREQ) checklist (Additional File 1).

The study was conducted in two hospitals in southern Saudi Arabia: a secondary care teaching hospital with 80 beds and a larger Ministry of Health hospital with 400 beds providing secondary and tertiary services. This selection captured variation in context, resources and services relevant to deprescribing. Ethical approval was obtained from the Scientific Research Ethical Committee at Najran University (443-41-60311-DS). Confirmation of approval was subsequently provided by the Queen’s University Belfast Faculty of Medicine, Health and Life Sciences Research Ethics Committee (MHLS 22_105).

### Sampling and recruitment

Inclusion and exclusion criteria were defined to focus the sample on HCPs directly involved in prescribing decisions for older hospitalised patients. Eligible physicians included residents, registrars, senior physicians, and consultants with clinical responsibility for older people. Consultants without routine involvement in older patients’ care were excluded. Eligible pharmacists required a ward-based role in prescribing decisions for older inpatients; those with less than two days per week of ward-based clinical pharmacy activities were excluded to ensure participants had consistent exposure to the ward’s clinical environment and multidisciplinary team (MDT) dynamics.

Participants were recruited using purposive sampling. Index contacts with prior or current relationships with one member of the research team (SA) suggested colleagues, who were then contacted by the researcher (TA) via email with study information. Those who expressed an interest in participation were sent a participant information sheet and consent form via email. Recruitment was guided by data saturation through a concurrent process of data collection and analysis.

### Data collection

Separate interview topic guides for physicians and pharmacists were developed by the research team, reflecting the TDF2 domains, with at least one question per domain (Additional File 2). The guides were piloted with two physicians and two pharmacists to assess clarity and interview duration; no amendments were required, and the pilot interviews were excluded from the analysis. Interviews focused on participants’ knowledge, experiences, and beliefs related to deprescribing. All interviews were conducted face-to-face in English or Arabic, depending on participants’ preference, with written informed consent obtained in advance.

Interviews were conducted by TA; a clinical pharmacist and research student trained in Good Clinical Practice and qualitative methods. Reflexivity was addressed through a diary in which TA recorded reflections after each interview. To enhance rigour, data were also independently analysed by a second researcher (CP), a female academic pharmacist with expertise in qualitative research.

### Data analysis

**Fig. 1:**
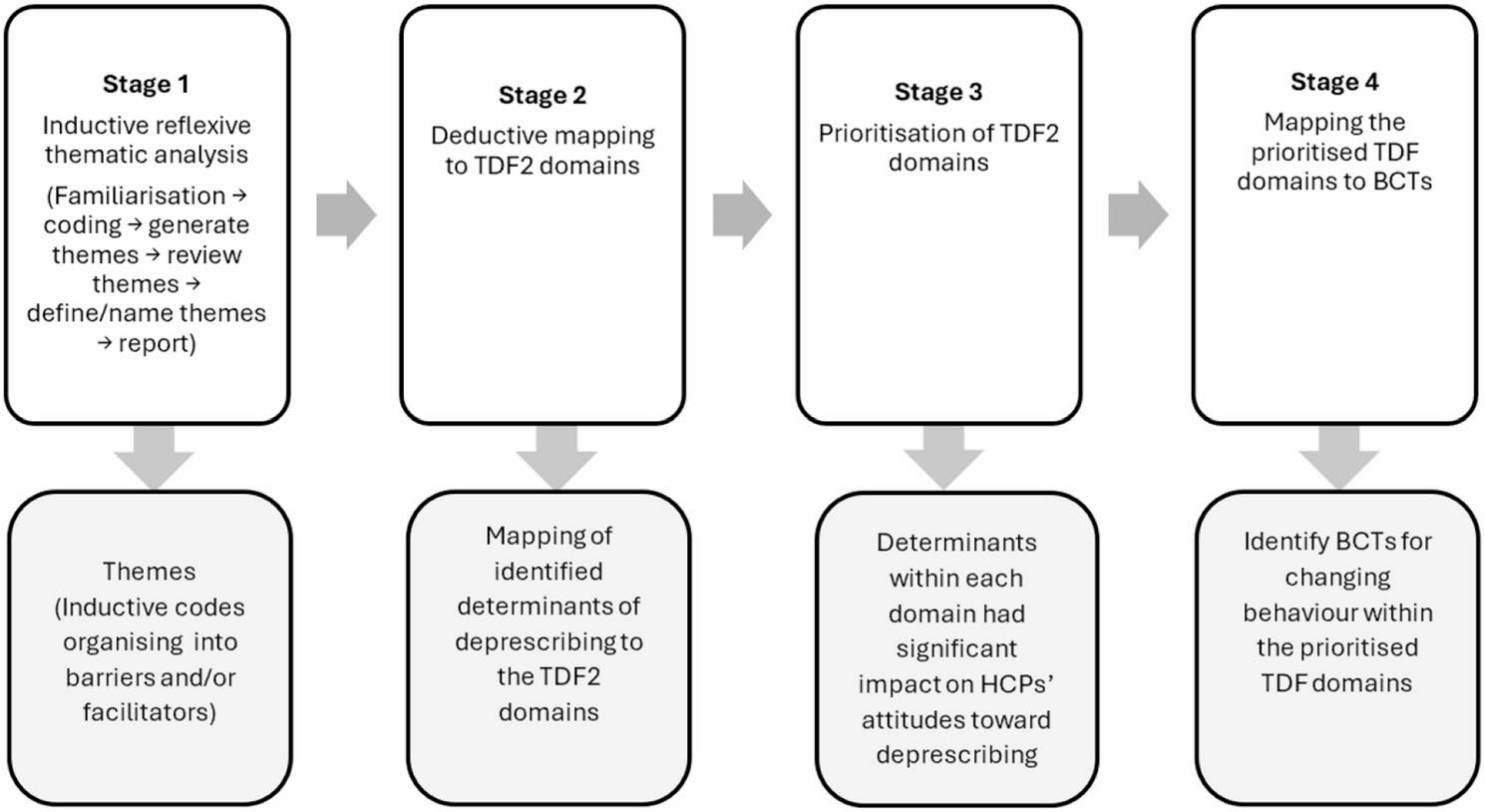
An overview of the four stages involved in data analysis

All interviews were audio-recorded, transcribed verbatim, and deidentified by TA. To ensure accuracy, each transcript was reviewed against the original recording by TA. Interviews conducted in Arabic were transcribed, then translated into English by TA and an external certified translation service. The two translations were compared to identify discrepancies, which were resolved via back-translation and discussion [27] ensuring accuracy and cultural integrity of the data. Data were managed and analysed using NVivo^®^ 12 (QSR International, Melbourne, Australia). Figure 1 provides an overview of these four stages with further details provided in the subsequent text.

#### Stage 1: Reflexive thematic analysis for identification determinants of deprescribing

Inductive reflexive thematic analysis was conducted using Braun and Clarke’s (2019) framework [28] to identify barriers to and facilitators of deprescribing. TA repeatedly listened to audio recordings, re-read transcripts and recorded initial impressions. Initial codes from five transcripts were independently developed by TA and CP, generating preliminary themes. After analysing these transcripts, TA and CP reviewed, named and discussed the themes and sub-themes, reaching consensus before progressing with the full analysis. Transcripts from physicians and pharmacists were coded separately and then combined into overarching themes.

#### Stage 2: mapping of identified determinants of deprescribing to the TDF2

TDF2 domain definitions guided the mapping process [25]. Coded data were organised as barriers, facilitators, or both. All inductive codes from Stage 1 were deductively mapped to relevant TDF2 domain(s).

#### Stage 3: prioritising TDF2 domains

Similar to previous studies [16, 29, 30], a domain was prioritised if the identified barriers or facilitators demonstrated strong influence on HCPs’ deprescribing attitudes in older people and were feasible to target within an intervention.

#### Stage 4: mapping prioritised TDF2 domains to BCTs

To identify potential intervention components, the prioritised TDF domains were mapped to specific BCTs using systematic matrices. These matrices functioned as a cross-referencing system, where a panel of experts linked specific techniques to the theoretical domains they are most likely to influence [31, 32]. Cane et al. (2015) [31] was used as the primary reference, as it included a larger number of BCTs and provided more comprehensive guidance for mapping. Michie et al. (2008) [32] was consulted to inform the identification of BCTs that had not been explicitly linked in the primary source.

## Results

### Sample

**Fig. 2:**
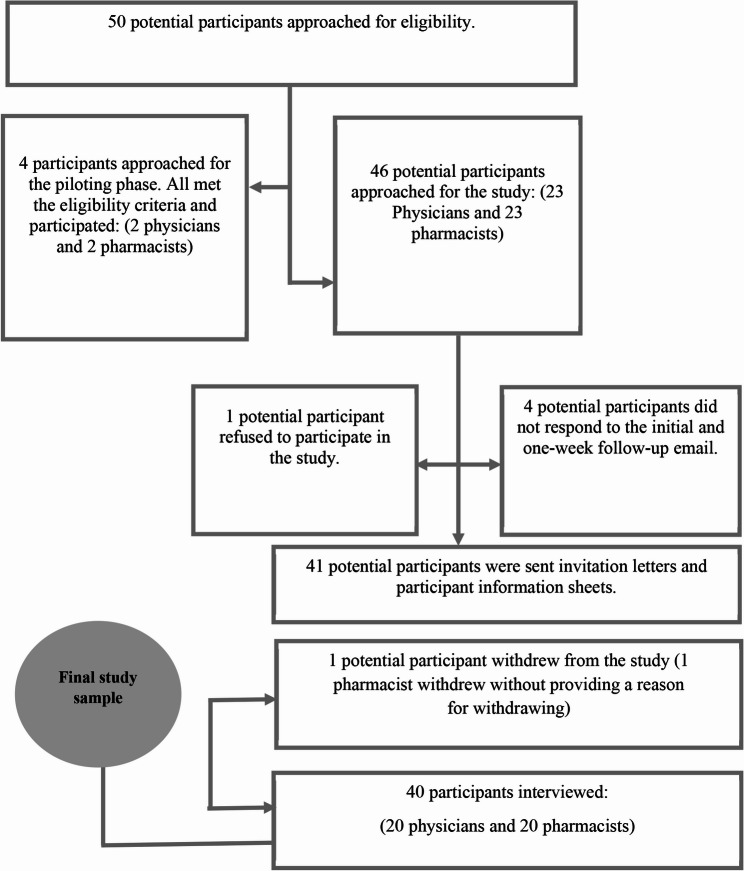
Participant recruitment process

A total of 50 potential participants from both groups were approached. Forty participants (20 physicians and 20 pharmacists) were interviewed between September 2022 and January 2023 (Fig. 2), at which point data saturation was considered to have been achieved across both groups. Half of the participants (nine physicians, 11 pharmacists) were interviewed in Arabic. Interviews lasted 40 ± 10.3 min, on average (range 40–55 min). Additional participant characteristics are presented in Table 1.

**Table 1:** Sample characteristics (*N* = 40)

Characteristic	Number (%) of participants
Gender	
Male	29 (73)
Female	11 (27)
Hospital	
Hospital 1^a^	22 (55)
Hospital 2^b^	18 (45)
Job title (physicians)	
Consultant	17 (85)
Specialist	2 (10)
General physician	1 (5)
Job title (pharmacists)	
Consultant clinical pharmacist	8 (40)
Senior clinical pharmacist	9 (45)
Inpatient pharmacist^c^	3 (15)
Years of professional experience	
1–5 years	9 (22)
6–10 years	27 (68)
> 10 years	4 (10)
Previously completed training or research projects on deprescribing	
Yes	9 (22)
No	31 (78)

^a^Teaching hospital

^b^Ministry of Health hospital

^c^In Saudi Arabia, hospital pharmacists are classified as inpatient, outpatient or clinical; only clinical pharmacists require a master’s degree and work in a specialty

### Stage 1: reflexive thematic analysis

Four themes were generated from the analysis, each encompassing barriers and facilitators of deprescribing in older people from the perspective of HCPs. The themes identified were:

#### Perceptions and consequences of deprescribing

Perceived risks and potential benefits were identified as key determinants of deprescribing. Many participants expressed concerns that discontinuing medications could lead to symptom recurrence.

*“If the patients need that medication*,* when we stop the medication… it can cause recurrence of symptoms.”* (Physician 6).

Conversely, participants recognised that deprescribing could optimise treatment regimens, reduce healthcare utilisation, and improve adherence and outcomes.

*“As we reduced unnecessary medications… we observed significant improvements… deprescribing helped reduce the pill burden… and this will be cost effective.”* (Physician 18).

*“*[Deprescribing can] *improve adherence… it is different if a patient takes 12 medications or two… In older patients*,* especially those living alone*,* adherence is a problem.”* (Pharmacist 2).

#### Roles of HCPs and communication

Pharmacists described their role in deprescribing as often limited by physicians’ authority to make final deprescribing decisions.

*“I can say our role as pharmacists; it is just you have to follow the physician’s prescriptions or advice.”* (Pharmacist 17).

In addition, physicians viewed themselves as responsible for initiating and leading the deprescribing process.

*“We as physicians should be at the forefront of deprescribing*,* ensuring that every medication has a clear purpose and that none are causing more harm than good.”* (Physician 7).

Improving interprofessional communication, adopting an MDT approach, and involving patients in deprescribing decisions were seen by participants as essential. Poor communication and fragmented care between services were described as barriers.

*“The most responsibility will be on the physicians… if there is collaborative* [working] *between the multidisciplinary team*,* this facilitates the deprescribing decisions.”* (Physician 18).

*“It’s critical to include patients in deprescribing discussions; it empowers them and often leads to better health outcomes.”* (Physician 19).

*“The communication breakdown between other hospitals and primary care is a challenge…medications might get continued simply because there was no clear communication about their initial purpose.”* (Pharmacist 5).

#### Factors influencing deprescribing decisions

Patient health status, comorbidities and treatment goals strongly influenced deprescribing decisions, with patients experiencing declining health often prioritised. However, some participants expressed hesitancy when life expectancy was limited.

*“Reducing medications is essential*,* particularly in multimorbid patients with declining health… we look at each drug critically.”* (Pharmacist 6).

Some participants expressed hesitancy when the clinical picture was unclear, citing perceived risks and uncertainty, while specialist knowledge enhanced confidence.

*“If the clinical picture is unclear*,* how can I know comorbidities and which medications to discontinue… the clinical picture is a crucial guide for deprescribing decisions.”* (Physician 3).

*“In my cardiology practice… I regularly review* [cardiovascular] *medications… if a patient is on furosemide… I feel confident to ask to stop or reduce the dose.”* (Pharmacist 4).

#### Culture, environment and resources

Participants highlighted poor integration between healthcare settings and limited access to complete medical records, delaying decision-making. Time pressures were also reported to limit opportunities for comprehensive medication reviews.

“*The factor that hinders me most is the lack of connection with other care facilities and access to systems… I want to deprescribe a medication but have no information in front of me.”* (Physician 16).

*“You know sometimes there is* [sic] *a lot of patients…admitted patients at the same time*,* 30 or 40 patients probably. I will not have a good chance to review all the medications.”* (Physician 6).

The lack of deprescribing guidelines and limited access to online resources further undermined clinicians’ confidence.

*“The online resources out there just aren’t enough to cover all the complexities we face in daily practice…the lack of guidelines makes it difficult.”* (Pharmacist 14).

Additionally, cultural attitudes and family influence often shaped patients’ acceptance of medication changes.

*“Patients don’t accept our plan and if we stop*,* they can visit another hospital and take this medication … cultural differences in health literacy which can affect a patient’s understanding of their medication and the reason for deprescribing.”* (Physician 5).

Participants recommended education and formal documentation of deprescribing outcomes to support quality improvement.

*“The medical team must have continuous education… a special training programme to emphasise deprescribing practice.”* (Pharmacist 3).

*“Having an outcome form would provide evidence to support my practice and ensure transparency.”* (Pharmacist 20).

### Stage 2: mapping of all identified determinants of deprescribing to the TDF2

Table 2 summarises how inductive codes were mapped to eight of the 14 TDF2 domains. These codes were categorised as barriers, facilitators, or both. All four themes were linked to multiple domains.

**Table 2:** Barriers to and facilitators of deprescribing in older adults from the perspectives of HCPs within each theme and linked to each of the TDF domains

Theme one: Perceptions and consequences of deprescribing		
Barriers inductive code	Facilitators inductive code	TDF domain
- Concern about recurrence of symptoms or withdrawal symptoms ^Doc, Ph^ - Deprescribing may lead to patient/carer complaints. ^Doc^	- Positive impacts of deprescribing including improved cognition, easier medication regimen (compliance), quality of life, decreased side effects (physical and mood), interactions and adverse effects. ^Doc, Ph^ - Reduced medication and health service expenditures. ^Doc, Ph^ - Medications which are not life-saving can be stopped. ^Doc, Ph^ - Some medication without benefits for older patients can be deprescribed, e.g. multivitamins and PPIs. ^Doc, Ph^	*Beliefs about* *consequences*
- Patient expectations and beliefs to have medication prescribed. ^Doc^ - Patient resistance to deprescribing. ^Doc, Ph^ - Pressure from carer and/or family to continue prescribing. ^Doc^ - Patients’ fear deprescribing. ^Doc^	- ND	*Social influences*
- ND	- Deprescribing improves physician satisfaction and professional recognition. ^Doc^	*Social/professional role and identity*

Theme two: Roles of HCPs and communication		
Barriers inductive code	Facilitators inductive code	TDF domain
- Difficult to communicate with older patients. ^Doc, Ph^	- Communication skills (e.g. training to improve communication with patients). ^Doc, Ph^ - Skills to convince physicians to accept pharmacist’s recommendation. ^Ph^	*Skills*
- Physicians may prescribe without knowing, monitoring or following up the patient. ^Ph^ - The role of the pharmacist is typically to recommend deprescribing to the physician. ^Ph^ - Deprescribing is the physician’s decision and responsibility and pharmacists cannot force physicians to deprescribe. ^Ph^ - Pharmacists don’t have authority to stop medicines or may not be their specialty. ^Ph^ - Physicians have the final decisions to deprescribe. ^Doc, Ph^ - Physicians have supremacy in terms of deprescribing responsibilities, while pharmacists have a limited role. ^Ph^	- Referring patients to another physician to deprescribe if outside participant’s area of specialism. ^Doc^ - Willing to deprescribe general medicines. ^Doc^ - Physicians are at the forefront of initiating the deprescribing process. ^Doc, Ph^	*Social/professional role and identity*
- Lack of trust between doctors and patients. ^Doc^ - Lack of trust between pharmacists and patients. ^Ph^ - Patients continue to take medicines they need against advice and can obtain medicines from a community pharmacy. ^Doc, Ph^ - Inadequate communication among prescribers across various healthcare settings. ^Doc, Ph^	- Teamwork with health professionals (inter-professional collaboration. ^Doc, Ph^ - Involvement of patients and carers in decision-making. ^Doc, Ph^	*Social influences*

Theme three: Factors influencing deprescribing decisions		
Barriers inductive code	Facilitators inductive code	TDF domain
- Not confident if clinical picture unclear. ^Ph^	- Confident deprescribing in limited life expectancy or multimorbid patients. ^Doc^ - Confident deprescribing medicines in own specialty. ^Doc, Ph^	*Beliefs about capabilities*
- “Risk of poor control of medical condition. ^Doc, Ph^ - Caution in deprescribing high risk medicines. ^Doc^ - Patients may not adhere to deprescribing. ^Doc, Ph^	- ND	*Beliefs about consequences*

Theme four: Culture, environment and resources		
Barriers inductive code	Facilitators inductive code	TDF domain
- Patients may take medicines prescribed by different providers. ^Doc, Ph^ - Incomplete medication history. ^Doc^ - Patients tend to visit multiple clinics with non-linked computer systems. ^Doc, Ph^ - Cultural issues with deprescribing for some patients. ^Doc, Ph^ - Deprescribing is limited in routine practice. ^Doc, Ph^	- Good history- taking for patients. ^Doc Ph^	*Environmental context/resources*
- Lack of a connected electronic system for cess across all clinics and hospitals. ^Doc, Ph^ - Time constraints – shortage of time in busy clinic and workload. ^Doc, Ph^ - Limited by lack of access to medical records and therapeutic plan. ^Ph^	- Use of prescribing appropriateness tools – Beers, STOPP/START. ^Doc^	*Environmental context/resources*
- Lack of local deprescribing guidelines for deprescribing. ^Doc, Ph^ - Lack of solid evidence to back recommendation. ^Doc, Ph^	- Knowledge of pharmacology, interactions, side-effects and clinical picture of patients. ^Doc,^ - Education - improved HCP awareness of deprescribing. ^Doc, Ph^	*Knowledge*
- Lack formal documentation for deprescribing. ^Doc, Ph^	- Monitor outcomes through frequent follow-up of lab results, medications and symptoms. ^Doc, Ph^	*Behavioural regulation*

*Doc* Doctor; *HCP* Healthcare Professional, *ND* Not Determined, *Ph* Pharmacist, *PPIs* Proton-pump inhibitors, *STOPP/START* Screening Tool of Older Person’s potentially Inappropriate Prescriptions and Screening Tool to Alert to Right Treatment criteria, *TDF* Theoretical Domains Framework

### Stage 3: prioritising TDF2 domains

A domain was prioritised if the identified barriers or facilitators within it demonstrated strong influence on HCPs’ deprescribing attitudes. Strong influence was determined by the strength of belief expressed by participants and convergence of views across both professional groups. Feasibility of targeting the domain within an intervention was also considered. Key barriers included that physicians have supremacy in deprescribing responsibilities, while pharmacists have a limited role; clinicians’ perceptions of negative patient and carer attitudes toward deprescribing; negative beliefs about outcomes; lack of guidelines; insufficient resources and poor connectivity between healthcare settings; and lack of formal documentation of deprescribing outcomes. Facilitators included interprofessional collaboration, education for both HCP groups and positive beliefs about outcomes. These were mapped to six TDF2 domains: *Social/professional role and identity*,* Social influences*,* Beliefs about consequences*, *Knowledge*, *Environmental context/resources* and *Behavioural regulation*.

### Stage 4: mapping the prioritised TDF domains to BCTs

Table 3 presents prioritised domains mapped to BCTs, which were identified to facilitate the implementation of deprescribing.

**Table 3:** Mapping of BCTs to key TDF domains including main barriers to and facilitators of deprescribing

Prioritised TDF domain	Determinants (barriers, facilitators) of deprescribing	Linked BCTs
*Social/professional role and identity* ^b^	Physicians have supremacy in terms of deprescribing responsibilities, while pharmacists have a limited role (barrier).	‘Social processes of encouragement, pressure, support’^,b^
*Social influences* ^a, b^	Negative patient and carer perceptions of deprescribing, social pressures that are not supportive of deprescribing (barrier). Inter-professional social support and collaboration between HCPs to encourage them to deprescribe (facilitator).	‘Information about others’ approval’^a^ ‘Restructuring the social environment’^a^ ‘Identification of self as role model’^a^ ‘Social comparison’^a^ ‘Vicarious reinforcement’^a^ ‘Modelling or demonstrating the behaviour^a^ / ‘Modelling/demonstration of behaviour by others’ ^b^ ‘Social support (unspecified) ^a^/ Social processes of encouragement, pressure, support’^b^ ‘Social support/ emotional’^a^ ‘Social support/ practical’^a^ ‘Social rewards’^a^
*Beliefs about* *consequences* ^a, b^	Negative beliefs about deprescribing outcomes (barrier). Positive beliefs about deprescribing outcomes (facilitator).	‘Information about emotional consequences’^a^ ‘Salience of consequences’^a^ ‘Social and environmental consequences’^a^ ‘Vicarious reinforcement’^a^ ‘Comparative imagining of future outcomes’^a^ ‘Pros and cons’^a^ ‘Covert conditioning’^a^ ‘Covert sensitization;^a^ ‘Anticipated regret’^a^ ‘Threat’^a^ ‘Persuasive communication’ ^b^ ‘Feedback’ ^b^ ‘Information regarding behaviour, outcome’ ^b^ ‘Self-monitoring of behaviour‘^b^
*Environmental* *context and resources* ^a, b^	Insufficient resources such as lack of IT resources, time constraint and lack of connections between different healthcare settings (barrier).	‘Restructuring the physical environment’^a^ ‘Prompts/cues^a^/ Environmental changes (e.g. objects to facilitate behaviour)’ ^b^ ‘Restructuring the social environment’^a^ ‘Discriminative (learned) cue’ ^a^ ‘Avoidance/changing exposure to cues for the behaviour’^a^
*Knowledge* ^a, b^	Education for both HCPs is perceived as facilitator for deprescribing (facilitator). Lack of deprescribing guidelines (barrier)	‘Information about health consequences^a^/ Information regarding behaviour, outcome ^b^ ‘Biofeedback’^a^ ‘Feedback on behaviour’^a^ ‘Antecedents’^a^
*Behavioural regulation* ^a, b^	Lack of formal documentation for deprescribing outcomes (barrier)	‘Self-monitoring of behaviour’^a^ ‘Goal/target specified: behaviour or outcome’ ^b^ ‘Contract’ ^b^ ‘Use of imagery’ ^b^ ‘Planning, implementation’ ^b^ ‘Prompts/triggers/cues’ ^b^

*BCT* Behaviour Change Technique, *TDF* Theoretical Domains Framework

^a^BCTs identified from Cane et al. (2015) [31]; ^b^BCTs identified from Michie et al. (2008) [32]

## Discussion

Deprescribing in Saudi hospitals is recognised as essential to optimising care for older people; however, its implementation is constrained by multiple determinants that reflect both international trends and distinct local healthcare dynamics [16–20]. This study extends current knowledge by applying the TDF2 to provide context-specific evidence, shifting the focus from a simple list of barriers to a systematic understanding of the behavioural drivers influencing clinical practice.

The findings indicate that, although physicians and pharmacists framed deprescribing as a shared responsibility, decision-making remained largely physician-led and pharmacists’ contributions were constrained by hierarchical dynamics. This was identified as a major barrier to deprescribing and reflects the *Social/professional role and identity* domain. Similar patterns have been reported in another study, where accountability for medication decisions can be deferred within clinical hierarchies (e.g., between junior and senior doctors), potentially delaying deprescribing initiation [33]. In this context, a “shared responsibility” narrative may not translate into action when decision authority and role expectations are not aligned [34]. Therefore, strengthening pharmacists’ involvement in ward-based medication review, clarifying their role in deprescribing discussions, and establishing mechanisms to document and escalate deprescribing recommendations may improve collaborative decision-making [35].

The *Beliefs about consequences* domain was a powerful influence on HCPs’ deprescribing behaviour. Consistent with previous research, observed positive outcomes following deprescribing appeared to reinforce clinicians’ confidence to deprescribe, whereas anticipated harms constrained engagement [9, 18, 20]. This uncertainty was compounded by gaps within the *Knowledge* domain. Participants valued training as an empowering factor, a finding that aligns with international evidence [17, 36, 37]. However, the absence of deprescribing-specific guidance and limited supporting evidence undermined clinicians’ ability to appraise and manage risk in complex polypharmacy. Given that developing deprescribing guidelines remains challenging and disease-specific guidelines may inadvertently contribute to polypharmacy [22, 38], these findings suggest that tailored, practical education should focus on translating evidence into actionable deprescribing processes, including structured risk–benefit appraisal and monitoring plans.

A critical finding of this study relates to the *Social influences* domain, specifically the perceptual gap between clinicians and patients. While participants identified multidisciplinary collaboration and active patient involvement as key facilitators in a manner consistent with existing evidence [17, 20], they simultaneously reported a high level of anticipated resistance from patients and carers. This perception contrasts with international evidence indicating that many patients and carers are open to deprescribing when clinicians recommend it [39–41]. This discrepancy suggests that HCPs may benefit from communication frameworks that help them navigate these conversations. Focusing on patient priorities and providing clear monitoring plans could help clinicians address their own concerns regarding patient attachment to long-term medications and fears of inadequate care [34].

System-level constraints significantly limit implementation. Poor connectivity and the absence of interoperable electronic health records between settings hinder continuity of care, leading to conflicting recommendations. These barriers align with findings from previous studies within the *Environmental context and resources* domain, which highlight that fragmented healthcare systems and high workloads are consistent impediments to deprescribing [16, 19, 20]. Critically, the systemic absence of documentation for outcomes remains a major impediment. Addressing this requires a focus on behavioural regulation, which refers to the formal procedures, such as monitoring and self-monitoring, intended to manage or change a behaviour. Without structured documentation of the rationale for deprescribing and a clear monitoring plan, the process lacks the continuity necessary for safe medication withdrawal [17, 25].

Identified TDF2 domains associated with factors influencing physicians’ and pharmacists’ deprescribing behaviour were mapped to 40 BCTs, offering a practical pathway for future hospital-based interventions in Saudi Arabia. Effective interventions should be multifaceted, designed to expand the pharmacist’s role in deprescribing, actively involve patients and carers in shared decision-making, and strengthen interprofessional communication within a more collaborative healthcare system. Furthermore, these interventions must prioritise education for HCPs and provide robust mechanisms for documentation and outcome monitoring to enhance the implementation of deprescribing.

A key strength of this study is its novel application of TDF2 within Saudi Arabian hospital settings, which provides a comprehensive and systematic account of the behavioural factors influencing deprescribing from the perspectives of physicians and pharmacists. This approach offers a structured foundation for designing targeted interventions. It strengthens the evidence base for culturally and administratively appropriate, context-specific deprescribing strategies in the region. However, the findings are based solely on the views of the physicians and pharmacists who participated in the study and may not reflect the perspectives of other HCPs involved in deprescribing. This may affect the transferability of the results to other settings or professional groups. In addition, the interviewer was a pharmacist, and participants were aware of this, which may have influenced their responses; however, this positionality was acknowledged in the analysis, thereby adding transparency and contextual depth to the findings. Patients and carers were not interviewed, and future studies should incorporate their perspectives to better inform patient-centred deprescribing and communication strategies.

## Conclusion

This study offers new insights into the challenges and opportunities for deprescribing in Saudi hospital settings. Building on these findings, the next step is to evaluate how the identified BCTs can be operationalised within a theory-based intervention to support the implementation of deprescribing for older people in this context.

## Supplementary Information

Supplementary Material 1.

Supplementary Material 2.

## Data Availability

Pseudonymised datasets generated and/or analysed during the current study are available from the corresponding author upon reasonable request.

## Abbreviations

BCTs: Behaviour Change Techniques
COREQ: Consolidated Criteria for Reporting Qualitative Research
HCPs: Healthcare Professionals
MDT: Multidisciplinary Team
PIMs: Potentially Inappropriate Medications
TDF2: Theoretical Domains Framework, Version 2
