# Supplementary material for: Barriers to and facilitators of deprescribing for older people in secondary care in Saudi Arabia: a qualitative study using a theory-based approach
Source: BMC Geriatr. 2026 Apr 14;26:729. doi: 10.1186/s12877-026-07486-8 (PMC13200391; doi:10.1186/s12877-026-07486-8)
Supplement: Supplementary file 1 — Supplementary Material 1. [file 12877_2026_7486_MOESM1_ESM.docx]

**Additional File 2** Interview topic guides (physicians and pharmacists)

**PHYSICIAN INTERVIEW TOPIC GUIDE**

**Introduction**

*“I’m Turkeah Alenzy, a PhD research student from the School of Pharmacy at QUB. Thank you for participating in this study. The purpose of this study is to explore healthcare professionals’ experiences and perspectives of deprescribing in older patients (65 years or older) in order to identify barriers to and facilitators of deprescribing. This will help in developing a future practitioner behaviour change intervention for enhancing deprescribing in the hospital setting. Deprescribing is an evidence-based medication review approach that focuses on monitoring, identifying, and discontinuing inappropriate medications. It has the potential to optimise the use of medications in this population, as well as to improve patients’ quality of life and reduce their risk of death. Please do not share the discussion with others outside of this interview. The discussion should last a maximum to one hour and will be audio-recorded. Direct quotations from the interview may be used in reports and papers, but confidentiality will be maintained, and it will not be possible to identify you in any publications or reports arising from this study. Participation is voluntary, and you may withdraw at any point during the interview without providing an explanation.*

*To start off, can you please confirm that you have read the information sheet that was emailed to you?*

*You have also electronically signed and returned the consent form. Can you confirm that you have read all of the information provided and you understand what you are being asked to do?*

*Are there any questions you’d like to ask me before we start?”*

[Turn the digital recorder on]

*“I’ll begin the interview by asking you some background questions about you. I’ll then ask you about deprescribing barriers, and facilitators. At the end of the interview, you will be asked to provide any additional information you believe is relevant for this study that has not been covered during the interview.”*

**Background information**

1. What is your clinical background and what qualifications do you have?
2. What is your current job title (resident, registrar, senior registrar physician or consultant)?
3. How long have you been practising as physician?
4. How long have you been practising in your current position?
5. Have you previously completed any training (either formal or self-directed) or research projects on deprescribing?
6. In your practice, what is the average number of regular medications prescribed per older patient (65 years or older)?

**Definitions**

*“A number of different definitions have been suggested for deprescribing. Some are simplistic, using deprescribing as a synonym for stopping drugs, whereas others include a variety of other factors and encompass various potential outcomes. For the purposes of this study, we will use the following definition for deprescribing:”*

[Hand interview participant a card with definition printed on it]

Deprescribing is defined as "the process of withdrawing an inappropriate medication while under the supervision of a health care professional with the goal of managing polypharmacy and improving outcomes". This has been expanded to incorporate the process of identifying and withdrawing a patient's medication when the benefit of taking the medication is outweighed by the potential harm. It includes dose reduction, tapering, or switching to a different class of medication while monitoring patient response.

Social /professional role and identity

- What do you consider your role/responsibilities to be as a physician in deprescribing for older patients?
- **Prompt:** Is there anything that you would consider to be beyond your contribution/ responsibility as a physician in deprescribing?
- **Prompt:** Who do you think is responsible for these aspects beyond your contribution/responsibility?

**Behavioural elicitation**

*“When we talk about deprescribing in older adults throughout the interview, it might be helpful to think of a situation where you have optimised prescribing or have deprescribed medicines for older people in your own practice. It may be useful to keep this example in mind. Don’t worry if you can’t think of a specific situation; just think about deprescribing in general terms using the definition I’ve just provided*.”

Knowledge

- What knowledge do you think you need as a physician to deprescribe medications for older people?
- **Prompt:** Is there any clinical knowledge in particular that you need?
- Specific knowledge sources/resources?
- Is there anything specifically relating to deprescribing for older patients?
- **Prompt:**  Do you think knowledge of guidelines (specific to deprescribing) is needed?
- Which guidelines?
- What evidence and/or guidelines pertaining to deprescribing for older patients are you familiar with or do you use in clinical practice?
- What do such guidelines recommend?
- **Prompt:** Do you think knowledge of the patient’s clinical picture is needed?
- What specific knowledge?
- In your view, which medications, if any, do not benefit older patients?
- What factors do you evaluate when deciding whether or not to deprescribe medications in older patients?

Skills

- What skills do you have as a physician that help you when deprescribing for older patients?
- **Prompt:** What skills help you interact with patients?
- **Prompt:** What skills help you interact with patients’ families?
- **Prompt:** What skills help you interact with other healthcare professionals?
- Is there any specific training which you feel would help you to approach deprescribing in older patients?

Beliefs about capabilities

- In what situations do you feel confident about deprescribing in older patients?
- **Prompt:** Why?
- In what situations do you not feel confident about deprescribing in older patients?
- **Prompt:** Why?
- **Prompt:** What might assist you in overcoming these difficulties?

Optimism

- How optimistic are you in medicines can be deprescribed’ for older patients?
- What would make you feel less optimistic that medicines can be deprescribed for older patients?
- **Prompt:** Why?

Beliefs about consequences

- What do you think are the benefits of deprescribing for older patients?
- **Prompt:** Think of positive long or short-term consequences for patients /their carers/ yourself/ the Health Ministry?
- What do you think are the potential risks associated with deprescribing for older patients?
- **Prompt:** Think of negative long or short-term consequences for patients /their carers/ yourself/ the Health Ministry?

Reinforcement

- What would encourage you to deprescribe medications in older patients?
- **Prompt:** Are there any incentives/rewards, e.g. personal rewards, professional recognition?
- **Prompt:** How do such incentives/rewards encourage you?
- What would prevent you from deprescribing medications in older patients?
- **Prompt:** Why?

Intentions

- How do you intend to address deprescribing for older patients?
- What would prevent you from deprescribing for older patients?
- **Prompt:** Why?

Goals

- To what extent is deprescribing medications a priority for you?
- **Prompt:** If low/high priority, why?
- Under what circumstances do you think it is less important to deprescribe medications for older patients?
- **Prompt:** Why?

Memory, attention and decision processes

- How do you usually remember to address deprescribing for older patients?
- **Prompt:** What do you do in practice (e.g. written records)?
- Under what circumstances might you forget to address deprescribing or find it difficult?
- **Prompt:** How would you describe the complexity of decision-making in deprescribing for older patients?

Environmental context and resources

- What resources or support might help you to deprescribe in older patients?
- **Prompt:** How do these help you?
- What are the factors that might hinder deprescribing in older patients?
- **Prompt:** Workplace environment and culture within practice, material resources available.

Social influences

- Who would influence your decision about deprescribing in older patients?
- **Prompts:** Patients; carers or relatives; colleagues in the hospital setting or other healthcare professionals.
- **Prompt:** Can you tell me more about what their influence is?

Emotion

- How does deprescribing in older patients make you feel?
- **Prompt:** Why does it make you feel this way?
- What effect might your own work-related stress or emotional engagement with an older patient and their carer have on your deprescribing decisions?

Behavioural regulation

- Having decided to deprescribe for an older patient, are there any ways in which you could monitor whether or not it has been done?
- What strategies could you use to monitor the outcomes of deprescribing for older patients?

**Prompt:** Outcomes may include improving medication appropriateness, enhancement of clinical outcomes

**Closing the interview**

*“That brings us to the end of the interview.*

*Is there anything else you feel has been left out of this discussion about deprescribing?*

*Do you have any additional comments about the interview’s content or format?*

*Thank you very much for making the time to speak with me today.”*[Turn the digital recorder off]

**PHARMACIST INTERVIEW TOPIC GUIDE**

**Introduction**

*“I’m Turkeah Alenzy, a PhD research student from the School of Pharmacy at QUB. Thank you for participating in this study. The purpose of this study is to explore healthcare professionals’ experiences and perspectives of deprescribing in older patients (65 years or older) in order to identify barriers to and facilitators of deprescribing. This will help in developing a future practitioner behaviour change intervention for enhancing deprescribing in the hospital setting. Deprescribing is an evidence-based medication review approach that focuses on monitoring, identifying, and discontinuing inappropriate medications. It has the potential to optimise the use of medications in this population, as well as to improve patients' quality of life and reduce their risk of death. Please do not share the discussion with others outside of this interview. The discussion should last a maximum to 40 minutes and will be audio-recorded. Direct quotations from the interview may be used in reports and papers, but confidentiality will be maintained, and it will not be possible to identify you in any publications or reports arising from this study. Participation is voluntary, and you may withdraw at any point during the interview without providing an explanation.*

*To start off, can you please confirm that you have read the information sheet that was emailed to you?*

*You have also electronically signed and returned the consent form. Can you confirm that you have read all of the information provided and you understand what you are being asked to do?*

*Are there any questions you'd like to ask me before we start?”*

[Turn the digital recorder on]

*“I'll begin the interview by asking you some background questions about you. I'll then ask you about deprescribing barriers, and facilitators. At the end of the interview, you will be asked to provide any additional information you believe is relevant for this study that has not been covered during the interview.”*

**Background information**

1. What is your clinical background and what qualifications do you have?
2. What is your current job title (e.g. second pharmacist, first pharmacist, clinical or consultant pharmacist)?
3. How long have you been practising as a pharmacist?”
4. How long have you been practising in your current position?
5. Have you previously completed any training (either formal or self-directed) or research projects on deprescribing?
6. In your practice, what is the average number of regular medications per older patient (65 years or older)?

**Definitions**

*“A number of different definitions have been suggested for deprescribing. Some are simplistic, using deprescribing as a synonym for stopping drugs, whereas others include a variety of other factors and encompass various potential outcomes. For the purposes of this study, we will use the following definition for deprescribing:”*

[Hand interview participant a card with definition printed on it]

Deprescribing is defined as "the process of withdrawing an inappropriate medication while under the supervision of a health care professional with the goal of managing polypharmacy and improving outcomes". This has been expanded to incorporate the process of identifying and withdrawing a patient's medication when the benefit of taking the medication is outweighed by the potential harm. It includes dose reduction, tapering, or switching to a different class of medication while monitoring patient response.

Social /professional role and identity

- What do you consider your role/responsibilities to be as a pharmacist in deprescribing for older patients?^*^
- **Prompt:** Is there anything that you would consider to be beyond your contribution/ responsibility as a pharmacist in deprescribing?
- **Prompt:** Who do you think is responsible for these aspects beyond your contribution/responsibility?

***Note:** The participant’s answer to this question will determine how subsequent questions are asked/worded (see below for sections shaded in grey)

**Behavioural elicitation**

*“When we talk about deprescribing in older adults for the remainder of the interview, it might be helpful to think of a similar situation where you have recommended optimising prescribing or deprescribing for older people in your own practice. This example may be useful keeping in mind. Don't worry if you can't think of a specific situation; just think about deprescribing in general terms using the definition I've provided.”*

Knowledge

- What knowledge do you think you need as a pharmacist when <recommending deprescribing, being involved in decisions to deprescribe or initiating deprescribing> for older people?
- **Prompts:** Is there any clinical knowledge in particular that you need?
- Specific knowledge sources/resources?
- Is there anything specifically relating to deprescribing for older patients?
- **Prompts:**  Is there any knowledge of guidelines (specific to deprescribing)

in particular that you need?

- What specific guidelines?
- What evidence and/or guidelines pertaining to deprescribing for older patients are you familiar with or do you use in clinical practice?
- What do such guidelines recommend?
- **Prompt:** Do you think knowledge of the patient’s clinical picture is needed?
- What specific knowledge?
- In your view, which medications, if any, do not benefit older patients?
- When you are asked to review an older patient’s medications, what factors do you evaluate when deciding whether or not to withdraw medications?

Skills

- What skills do you have as a pharmacist that would enable you to <recommend deprescribing, be involved in decisions to deprescribe or initiate deprescribing> in older patients?
- **Prompt:** What skills help you interact with patients?
- **Prompt**: What skills help you interact with patients’ families?
- **Prompt**: What skills help you interact with other healthcare professionals?
- Is there any specific skills training which you feel would help you to <recommend deprescribing, be involved in decisions to deprescribe or initiate deprescribing> in older patients?

Beliefs about capabilities

- In what situations do you feel confident when <recommending deprescribing, being involved in decisions to deprescribe or initiating deprescribing>in older patients?
- **Prompt:** Why?
- In what situations do you not feel confident when <recommending deprescribing, being involved in decisions to deprescribe or initiating deprescribing>in older patients?
- **Prompt:** Why?
- **Prompt:** What might assist you in overcoming these difficulties?

Optimism

- How optimistic are you that medicines can be deprescribed for older patients?
- What would make you feel less optimistic in medicines can be deprescribed for older patients?
- **Prompt:** Why?

Beliefs about consequences

- What do you think are the benefits of deprescribing for older patients?
- **Prompt:** Think of positive long or short-term consequences for patients /their carers/ yourself/ the Health Ministry?
- What do you think are the potential risks associated with deprescribing for older patients?
- **Prompt:** Think of negative long or short-term consequences for patients /their carers/ yourself/ the Health Ministry?

Reinforcement

- What would encourage you to <recommend deprescribing, be involved in decisions to deprescribe or initiate deprescribing>in older patients?
- **Prompt:** Are there any incentives/rewards, e.g. personal rewards, professional recognition?
- **Prompt:** How such things incentives/rewards encourage you?
- What would prevent you to <recommend deprescribing, be involved in decisions to deprescribe or initiate deprescribing>in older patients?
- **Prompt:** Why?

Intentions

- How do you intend to address <recommending deprescribing, being involved in decisions to deprescribe or initiating deprescribing>in older patients?
- What would prevent you from addressing <recommending deprescribing, being involved in decisions to deprescribe or initiating deprescribing>in older patients?
- **Prompt:** Why?

Goals

- To what extent is <recommending deprescribing, being involved in decisions to deprescribe or initiating deprescribing>in older patients?a priority for you?
- **Prompt:** If low/high priority, why?
- Under what circumstances do you think it is less important to deprescribe medications?
- **Prompt:** Why?

Memory, attention and decision processes

- How do you usually remember to address issues with <recommending deprescribing, being involved in decisions to deprescribe or initiating deprescribing>in older patients?
- **Prompt:** What do you do in practice (e.g. written records)?
- Under what circumstances might you forget or find it difficult to address these issues?
- **Prompt:** How would you describe the complexity of decision-making in <recommending deprescribing, being involved in decisions to deprescribe or initiating deprescribing>in older patients?

Environmental context and resources

- What resources or support might help you to <recommend deprescribing, be involved in decisions to deprescribe or initiate deprescribing>in older patients?
- **Prompt:** How do these help you?
- What are the factors that might prevent you from <recommend deprescribing, be involved in decisions to deprescribe or initiate deprescribing>in older patients?
- **Prompt:** Workplace environment and culture within practice, material resources available.

Social influences

- Who would influence your decisions to <recommend deprescribing, be involved in decisions to deprescribe or initiate deprescribing>in older patients?
- **Prompt:** Patients; carers or relatives; colleagues in the hospital setting or other healthcare professionals.
- **Prompt:** Can you tell me more about what their influence is?

Emotion

- How does <recommending deprescribing, being involved in decisions to deprescribe or initiating deprescribing>in older patients make you feel?
- **Prompt**: Why?
- What effect might your own work-related stress or emotional engagement with an older patient and their carer have on your decisions around <recommending deprescribing, being involved in decisions to deprescribe or initiating deprescribing>?

Behavioural regulation

- Having decided to <recommend deprescribing, be involved in decisions to deprescribe or initiate deprescribing>for older patients, are there any ways that could monitor whether or not it has been done?
- What strategies could you use to monitor the outcomes when you <recommend deprescribing, be involved in decisions to deprescribe or initiate deprescribing>for older patients?
- **Prompt:** Outcomes may include improving medication appropriateness, enhancement of clinical outcomes

**Closing the interview**

“*That brings us to the end of the interview.*

*Is there anything else you feel has been left out of this discussion about deprescribing?*

*Do you have any additional comments about the interview's content or format?*

*Thank you very much for making the time to speak with me today.”* [Turn the digital recorder off]
