# Supplementary material for: Barriers to and facilitators of deprescribing for older people in secondary care in Saudi Arabia: a qualitative study using a theory-based approach
Source: BMC Geriatr. 2026 Apr 14;26:729. doi: 10.1186/s12877-026-07486-8 (PMC13200391; doi:10.1186/s12877-026-07486-8)
Supplement: Supplementary file 2 — Supplementary Material 2. [file 12877_2026_7486_MOESM2_ESM.docx]

**Additional File 1** Consolidated criteria for reporting qualitative research (COREQ) checklist

| **Topic** | | **Item No.** | **Guide Questions/Description** | **Reported on**  **Page No.** |
| --- | --- | --- | --- | --- |
| **Domain 1: Research team and reflexivity** | | | | |
| *Personal characteristics* | | | | |
| Interviewer/facilitator | | 1 | Which author/s conducted the interview or focus group? | 2 and 7 |
| Credentials | | 2 | What were the researcher’s credentials? e.g. PhD, MD | N/A |
| Occupation | | 3 | What was their occupation at the time of the study? | 7 |
| Gender | | 4 | Was the researcher male or female? | 7 |
| Experience and training | | 5 | What experience or training did the researcher have? | 7 |
| *Relationship with participants* | | | | |
| Relationship established | | 6 | Was a relationship established prior to study commencement? | N/A |
| Participant knowledge of  the interviewer | | 7 | What did the participants know about the researcher? e.g. personal  goals, reasons for doing the research | 6 |
| Interviewer characteristics | | 8 | What characteristics were reported about the interviewer/facilitator?  e.g. Bias, assumptions, reasons and interests in the research topic | N/A |
| **Domain 2: Study design** | | | | |
| *Theoretical framework* | | | | |
| Methodological orientation and Theory | | 9 | What methodological orientation was stated to underpin the study? e.g. grounded theory, discourse analysis, ethnography, phenomenology,  content analysis | 2 and 7 |
| *Participant selection* | | | | |
| Sampling | | 10 | How were participants selected? e.g. purposive, convenience,  consecutive, snowball | 2 and 6 |
| Method of approach | | 11 | How were participants approached? e.g. face-to-face, telephone, mail,  email | 7 |
| Sample size | | 12 | How many participants were in the study? | 2 and 9 |
| Non-participation | | 13 | How many people refused to participate or dropped out? Reasons? | 9; Figure 2 |
| *Setting* | | | | |
| Setting of data collection | | 14 | Where was the data collected? e.g. home, clinic, workplace | 6 and 7 |
| Presence of non-  participants | | 15 | Was anyone else present besides the participants and researchers? | No |
| Description of sample | | 16 | What are the important characteristics of the sample? e.g. demographic  data, date | 10; Table 1 |
| *Data collection* | | | | |
| Interview guide | | 17 | Were questions, prompts, guides provided by the authors? Was it pilot  tested? | 6 |
| Repeat interviews | | 18 | Were repeat interviews carried out? If yes, how many? | N/A |
| Audio/visual recording | | 19 | Did the research use audio or visual recording to collect the data? | 7 |
| Field notes | | 20 | Were field notes made during and/or after the interview or focus group? | 6 |
| Duration | | 21 | What was the duration of the interviews or focus group? | 9 |
| Data saturation | | 22 | Was data saturation discussed? | 8 |
| Transcripts returned | | 23 | Were transcripts returned to participants for comment and/or correction? | No |
| **Domain 3: analysis and findings** | | | | |
| *Data analysis* | | | | |
| Number of data coders | 24 | | How many data coders coded the data? | 8 |
| Description of the coding  tree | 25 | | Did authors provide a description of the coding tree? | 8 |
| Derivation of themes | 26 | | Were themes identified in advance or derived from the data? | 8 |
| Software | 27 | | What software, if applicable, was used to manage the data? | 7 |
| Participant checking | 28 | | Did participants provide feedback on the findings? | No |
| *Reporting* | | | | |
| Quotations presented | 29 | | Were participant quotations presented to illustrate the themes/findings?  Was each quotation identified? e.g. participant number | 10-14 |
| Data and findings consistent | 30 | | Was there consistency between the data presented and the findings? | Refer to the Results section. We sought to present the study outcomes with clarity and consistency, ensuring they accurately represent the data obtained. |
| Clarity of major themes | 31 | | Were major themes clearly presented in the findings? |  |
| Clarity of minor themes | 32 | | Is there a description of diverse cases or discussion of minor themes? |  |
